# Expansion of the tmRNA sequence database and new tools for search and visualization

**DOI:** 10.1093/nargab/lqaf019

**Published:** 2025-03-18

**Authors:** Eric P Nawrocki, Anton I Petrov, Kelly P Williams

**Affiliations:** Division of Intramural Research, U.S. National Library of Medicine, National Institutes of Health, Bethesda, MD 20894, United States; Riboscope Ltd, 23 King Street, Cambridge CB1 1AH, United Kingdom; Sandia National Laboratories, Livermore, CA 94550, United States

## Abstract

Transfer–messenger RNA (tmRNA) contributes essential tRNA-like and mRNA-like functions during the process of *trans*-translation, a mechanism of quality control for the translating bacterial ribosome. Proper tmRNA identification benefits the study of *trans*-translation and also the study of genomic islands, which frequently use the tmRNA gene as an integration site. Automated tmRNA gene identification tools are available, but manual inspection is still important for eliminating false positives. We have increased our database of precisely mapped tmRNA sequences over 50-fold to 97 179 unique sequences. Group I introns had previously been found integrated within a single subsite within the TψC-loop; they have now been identified at four distinct subsites, suggesting multiple founding events of invasion of tmRNA genes by group I introns, all in the same vicinity. tmRNA genes were found in metagenomic archaeal genomes, perhaps a result of misbinning of bacterial sequences during genome assembly. With the expanded database, we have produced new covariance models for improved tmRNA sequence search and new secondary structure visualization tools.

## Introduction

Transfer–messenger RNA (tmRNA) is a bacterial RNA with both tRNA-like and mRNA-like properties. When the bacterial ribosome reaches the end of an incomplete mRNA lacking a stop codon, tmRNA becomes engaged in the process of *trans*-translation, in which the truncated protein product first receives the alanyl moiety charging the tmRNA and then a proteolysis-inducing peptide tag encoded in tmRNA [1]. This leads to degradation of the truncated protein, speeded degradation of the incomplete mRNA, and freeing of the stalled ribosome [2]. tmRNA occurs with its dedicated protein cofactor SmpB [3] in nearly all bacteria, and in some mitochondria and plastids [4–9].

**Figure 1.**
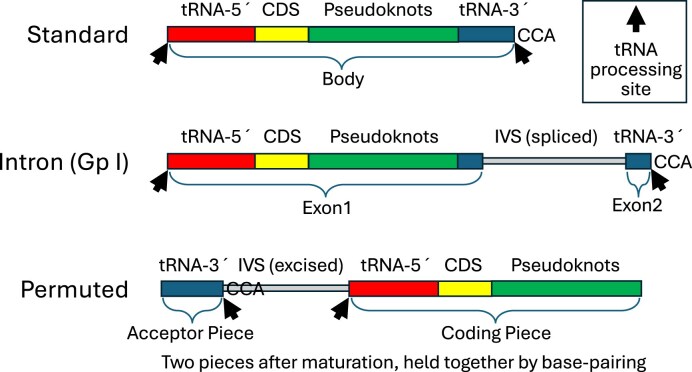
Three main tmRNA gene forms and their key segments. CDS: tag coding sequence. IVS: intervening sequence absent in mature tmRNA. CCA: position corresponding to the CCA tail, not always encoding CCA.

Three major gene forms are known (Fig. 1): (i) standard [10]; (ii) intron, interrupted in the TψC-loop region by a self-splicing group I intron [11], and (iii) permuted, a circularly permuted gene form whose transcript is cleaved by tRNA processing enzymes into a two-piece mature tmRNA held together by base pairing [8]. A comprehensive database of tmRNA genes is useful for the study of *trans*-translation, but also for annotating genomes, to help map genomic islands that often integrate into the tmRNA gene [12], and for the study of pseudoknots that typically number four per tmRNA.

We have increased the previous database [13], whose website is no longer maintained (see the “Data availability” section), over 50-fold to 97 179 unique curated sequences. With this new information, we have produced new covariance models (CMs), two of which are available at the Rfam database [14], and demonstrated their utility for tmRNA gene search. We have also updated the secondary structure visualization software R2DT [15] and prepared R2DT templates tailored to the various tmRNA gene forms.

## Materials and methods

### Genomes

The following sets of genomic assembly sequences were collected from the National Center for Biotechnology Information (NCBI): 451 630 bacterial and 8305 archaeal genomes (Supplementary Table S1), RefSeq [16] viruses downloaded on 6 October 2023, mitochondrial/plastid genomes downloaded on 9 September 2023, and 6 chromatophore genomes downloaded on 27 October 2023.

### Bacterial and archaeal taxonomy

Genomes were assigned to species of the GTDB taxonomy system [17], release 214, as follows. Many had already been assigned by GTDB. For the remainder, the program Speciate [18] was applied to attempt assignment to a GTDB species representative, adding representatives for four species (*Hodgkinia cicadicola*, *Carsonella ruddii*, *Tremblaya princeps*, and *Nasuia deltocephalinicola*) that are systematically excluded from GTDB.

Some genomes (2780) did not meet the GTDB-specified criteria for species assignment (usually 95% average nucleotide identity to a representative). These were first assigned to domain and then to finer taxonomic rank as follows. Pre-existing alignments of key protein families (53 for Archaea, 120 for Bacteria) for species representatives were taken from GTDB. For each of the five shared families (PF00410.20, PF00466.21, TIGR00064, TIGR00967, and TIGR01171), the archaeal and bacterial GTDB alignments were merged using MAFFT v. 7 [19] (e.g. mafft --merge subMSAtable TIGR00064.premerge > TIGR00064.merge).

Protein sequences for the unassigned genomes were taken using Prodigal v. 2.6.1 [20] and the key conserved family hidden Markov models (HMMs) were applied using HMMER v3.1b2 (http://hmmer.org/) (e.g. hmmsearch --cpu 0 --domtblout gtdb.domtbl 168.hmm protein.faa), taking the top hit above cutoff for each HMM. To assign domain, new proteins from the five shared families were added to each GTDB alignment (e.g. mafft --add newTIGR00064.fa gtdbTIGR00064.afa > allTIGR00064.afa). The five alignments were concatenated, and a tree prepared using FastTree v. 2.1.9 [21] with the LG substitution matrix.

For each unassigned genome, the smallest clade that contained at least one reference genome was used to assign a domain, yielding 346 assignments to Archaea and 2434 to Bacteria. For finer taxonomic assignment, separately for archaeal and bacterial protein family sets, the new sequences were added to the GTDB alignments, which were concatenated, and trees were prepared as above. Taxonomic assignment was based on the smallest clade that contained at least one reference genome. This process assigned 1626 of the 2780 genomes to species, 719 to genus, 270 to family, 63 to order, 40 to class, 43 to phylum, and 19 to domain. The taxonomic labels for these originally unassigned genomes were marked by appending ‘__X’ and a serial number for that label.

### tmRNA gene search phase 1: major batches

Three automated tmRNA-finding tools were applied to batches of genomes: (i) Aragorn v. 1.2.40 [22], (ii) five tmRNA CMs from Rfam [14] (RF00023, RF01849, RF01850, RF01851, and RF02544), applied using Infernal v. 1.1.2 [23], and (iii) BLASTN [24] against the then-current version of our tmRNA database, as managed by our software Rfind [25]. Perfect matches to existing database sequences were temporarily discarded. Based on the scores, hits were designated “S” (strong: Aragorn >103 or Infernal bit score >140 or Rfind >90) or otherwise “W” (weak). Because the CMs yielded many false positives, many of which were to tRNAs, the main protocol for tmRNA gene search used only Aragorn and Rfind hits, collecting the novel hit genomic regions with their 250-bp flanks. Sequences likely to be tRNA genes were identified as follows and rejected. tRNA genes in the genome were identified using tRNAscan-SE v. 2.0 [26] as managed by tFind software [27]. tmRNA assignments were rejected if overlapped for >5 bp by a tRNA scoring >42 bits, unless the gene form was permuted and the hit was strong. If there was an Rfind hit only, and the match to the reference genome was truncated within mature tmRNA sequence at a contig end, the assignment was rejected.

Preliminary mapping of gene segments (Fig. 1) was taken from Aragorn output if available, otherwise from the expected segment boundary positions based on the best BLASTN match to a database reference. For each of the three gene forms, old database and new candidate sequences were chunk-aligned, meaning that preliminary segment boundaries were aligned, and segments were expanded at their centers with dashes to reach the length of the longest instance of the segment. The chunk-aligned sequences were arranged in taxonomic order to enable comparative analysis of segment boundaries, and their labels were preceded with their “S” or “W” designation as above. In a manual phase, the three gene form alignments were adjusted to optimize segment boundaries, and more assignments were rejected.

All new sequences in each alignment were visually inspected (SeaView v. 5.0.4) [28], beginning with the region of the most conserved segment, tRNA-like-3′, spending less time on weak assignments; this first pass enabled many rejections, although search to the left or right sometimes found the segment in a shifted position. Inspection continued at the tRNA-like-5′ segment, and other segment boundaries, sometimes requiring translation of the CDS region to mark its boundaries; frameshifts were allowed in the CDS when resume and stop codons could be validated comparatively.

Special attention was required at the extreme ends of permuted genes and at the group I intron boundaries. Additional rejections were triggered by (i) large internal blocks of ambiguous bases or (ii) large internal deletions found in certain older metagenome-assembled genomes (MAGs) affecting the tRNA or CDS portions, identified by comparison to related sequences. Additional truncated forms were identified and rejected in the manual phase, insisting that sequences include all three positions equivalent to the tRNA CCA tail.

Consideration was also given to potentially miscalled forms; for example, several intron forms were initially miscalled because the best BLASTN hit was to a standard-form reference. After manual realignment, flanking sequences were trimmed, followed by deduplication. Three final checks were automated: (i) Sequences with a block of two or more ambiguous bases were rejected. (ii) Cases where the 7-bp acceptor stem had two or more mismatches were identified, and later either corrected by shifting the tRNA-like-5′ segment or rejected. (iii) Cases where the tag reading frame had an internal stop codon or no terminal stop codon were identified, and later corrected by shifting the alignment (although a small number with apparent frameshifts or internal/terminal stop codons were noted and retained). The above process has been iterated approximately biannually in four major batches as our bacterial/archaeal genome collection has grown since 2016; statistics for the latest major batch update are given in Table 1. The process was also applied to the organellar and viral genomes.

**Table 1.** Statistics for processing of bacterial/archaeal sequences in the latest major batch update to the database

Previous unique database sequences	73 023
New genomes as of GTDB r214	62 217
Aragorn/Rfind hits to new genomes	75 566
After skipping identical to previous	50 911
After rejecting likely tRNAs	38 739
After deduplication	34 719
After rejecting truncations	27 992
After manual inspection, sanity checks	23 175
Total after latest major batch	96 079

### tmRNA gene search phase 2: special search in under-represented taxa

After the latest major update batch, an additional phase of tmRNA finding was undertaken for bacterial taxa with low tmRNA gene yields, choosing candidates based on Rfam data (in the above work, candidates had been selected based on Aragorn or BLASTN data only). All 111 non-nested bacterial taxa were identified that had at least 20 genomes with failed tmRNA identification in >50% of the genomes. For each of the 4590 genomes in these taxa where tmRNA had not yet been identified, a single top candidate tmRNA sequence was taken based on the best Rfam hit for the genome. For each taxon separately, the known tmRNA sequences and new candidates were subjected to the above tmRNA search process. Numerous (2080) candidates were rejected as being tRNAs; many remaining candidates were rejected as truncated sequences or for the other reasons noted above, leaving 763 valid instances that added 467 unique sequences to the database. After the three phases of gene search, each unique sequence was named according to the species where it was most frequent, followed by a period and a serial number for abundance rank within that species.

### tmRNA gene search phase 3: special search for new introns

The new CM tmRNA_intron (see the “New covariance models” section) was applied to all 33 537 bacterial genomes missing a tmRNA assignment, and cutoffs were applied that excluded all non-intron tmRNA sequences, but were nonetheless conservative: a size cutoff of 500 bp (the smallest known intron form was 545 bp) together with a bit score cutoff of 150 (the lowest known intron bit score was 225.3). This yielded 42 new unique sequences, to which were added three missing mitochondrial sequences [29], for a final count of 97 179 sequences.

### New covariance models

We took advantage of the diversity of new tmRNA genes discovered in this work to update the Rfam tmRNA CMs. We present three new CMs: tmRNA (standard form), tmRNA_permuted, and tmRNA_intron. The standard model is an update of the Rfam RF00023 CM, which was built from a “seed” alignment of 477 sequences, unchanged since Rfam 1.0. We used the existing RF00023 model to align all standard tmRNA sequences found by our pipeline using the command “cmsearch --rfam -T 50 -A,” merged that alignment with the existing RF00023 seed, and then filtered to 60% identity using the esl-weight program (“esl-weight -f --idf 0.60”) to yield a 3038-sequence alignment, which was refined using “cmbuild --refine” to generate the final alignment and model.

For the permuted form, we merged the three Rfam 15.0 models alpha_tmRNA (RF01849), beta_tmRNA (RF01850), and cyano_tmRNA (RF01851) models into a single model named tmRNA_permuted, to enable simpler and faster identification of these sequences with Rfam. When the three existing models, which capture the diversity and secondary structure of permuted tmRNAs in three different bacterial groups (Alphaproteobacteria, Betaproteobacteria, and Cyanobacteria), were added to Rfam in release 10.1 [5], it was not clear that a single model could recognize all sequences from all three divisions. The abundance of new and diverse sequences identified in our pipeline facilitated the construction of a merged model capable of robustly identifying all three types. The merged model was created by first combining the alpha and beta sequences into a single alignment and then merging the cyano sequences. The set of seed sequences was selected by filtering alignments of all hits in the database to each of the three existing Rfam permuted families to roughly 100 sequences each: 97 alphaproteobacterial sequences, filtered to 57.5% identity; 101 betaproteobacterial sequences, filtered to 88% identity; and 98 cyanobacterial sequences, filtered to 92% identity. We aligned these 296 sequences to our merged model and used that alignment as input to “cmbuild --refine” (Infernal v. 1.1.5) to create our final alignment and model. The new tmRNA_permuted model contains consensus base pairs only from the tRNA domain, which are shared by all three existing Rfam models, and does not include the protruding 5′ or 3′ terminal regions. The new tmRNA_permuted model (new Rfam CM RF04321) was able to identify all permuted sequences, which none of the three existing permuted Rfam models could achieve.

To create the tmRNA_intron model, which has no cognate in Rfam, we started with an alignment of the 844 intron-containing tmRNA sequences initially identified by our pipeline. The group I intron region of the alignment was manually refined, marking the canonical stems, and was then filtered using the “esl-weight” program to 70% identity. We used Pfam and HMMER to detect homology to LAGLIDADG homing endonuclease protein-coding genes (HEGs) in 23 of the 844 introns, and added any of these 23 removed by the 70% filtering back into the alignment, to enable the model to better detect tmRNAs with long introns that encode HEGs, leaving 98 sequences in the final alignment used to build the CM.

The updated standard tmRNA and new tmRNA_permuted Rfam families will be available as of release Rfam 15.1. It is currently not possible to include the tmRNA_intron model in Rfam due to the requirement that a family can belong to only one “clan”. Clans in Rfam represent groups of homologous families that allow overlapping hits, and the tmRNA_intron model would need to be part of both the tmRNA clan (CL00001) and a group I intron clan, which would include the Intron_gpI family (RF00028). This requirement is integral to Infernal’s process for distinguishing overlapping hits within and outside of clans when using the Rfam library of CMs. Future developments in Infernal may enable the inclusion of the tmRNA_intron model in Rfam. In the meantime, the intron model is provided alongside the standard and permuted tmRNA models, and the alignments used to build them, as Supplementary File S1.

### New R2DT templates

Six new R2DT templates were generated using the standard procedure outlined in the R2DT documentation (https://docs.r2dt.bio/en/latest/templates.html): “tmRNA” (based on the updated RF00023 model), “tmRNA_intron” (based on the intron model presented in this work), and “tmRNA_alpha,” “tmRNA_beta,” “tmRNA_cyano,” and “tmRNA_mito” (based on the Rfam 15.0 models RF01849, RF01850, RF01851, and RF02544, respectively). Starting points for the templates were generated for each of the six models by inputting the model consensus sequence (generated using Infernal’s “cmemit” program with the “-c” option) and consensus secondary structure in dot-parentheses format into the R2DT server (https://rnacentral.org/r2dt), and then saving the generated structure layout in JSON format. Each layout was then input and manually edited using RNAcanvas [30] to ensure that the arrangement of structural elements was consistent with the usual tmRNA secondary structure commonly found in the literature [8, 10, 31].

## Results and discussion

Our previously published tmRNA database included sequences that were incomplete or contained numerous ambiguous bases [13]. Now we exclude such lower quality sequences and allow only complete genes found in genome projects available at NCBI. Since the automated tools (Aragorn [22], Rfind [25], and application of tmRNA CMs with Infernal [23]) can yield false positives or sequences that do not meet our newer criteria, we have deemed it necessary to visually inspect all new candidate sequences in an alignment editor. Although this aspect of the major batch searches is assisted by automated preprocessing of sequences and followed by automated sanity checks, it is nonetheless largely manual. After performing four major updates since 2016, and two specialized searches, our current database contains 97 179 unique tmRNA gene sequences, all complete from the 5′ to 3′ end, except for eight plastidial sequences of taxonomic interest that are missing terminal sequences because they were derived by polymerase chain reaction. Tables 2 and 3 show taxonomic and form breakdowns.

**Table 2.** High-level taxonomic breakdown of unique tmRNA sequences

Taxonomic group	Sequences
Bacteria	96 539
Plastids	416
Bacteriophage	79
Mitochondria	73
Archaea	68
Chromatophores	4
Total	97 179

The bacterial source was favored in cases where the identical sequence was found in bacterial and archaeal genomes. See Supplementary Table S2 for phylum-level breakdown.

**Table 3.** Unique sequence counts for each form

Form	Count
Standard	84 269
Permuted	11 946
Intron	883
Permuted, no CDS	72
Standard, no CDS	9
Total	97 179

See Supplementary Table S2 for taxonomic breakdown.

An unexpected finding is that there were 73 unique tmRNA genes (in 78 instances) found in archaeal genomes; no archaeon has previously been known to produce tmRNA. It would be exciting if tmRNA function in archaea could be proven, or even if archaea sporadically capture this gene from bacteria without being able to use it. However, one null hypothesis is that these are artifacts from misbinning of bacterial contigs during metagenomic assembly. All these genome assemblies were marked “derived from metagenome” at NCBI. Furthermore, five of these genes were identical to bacterial ones, and the remaining unique sequences were inconsistent in both taxonomic distribution (Supplementary Table S2) and form (1 intron, 19 permuted, 48 standard). The tmRNA gene (Methanofastidiosum__sp012799835.1) of intron form showed close sequence relationships to those of the bacterial genus *Tissierella*, in both its intron and tmRNA portions, and not to any from a database of archaeal group I introns. We therefore cannot rule out that Methanofastidiosum__sp012799835.1, nor any of the archaeal tmRNAs, is bacterial, misbinned into an archaeal MAG. Such MAG misbinning may also cause other less obvious (within-Bacteria) phylogenetic discrepancies; 94 of our sequences have bacterial cross-phylum instances. A unique feature found in one of these nominally archaeal tmRNAs (permuted GCA-2688965__sp002688965.2) is an intact, high-scoring tRNA gene (tRNA-Ala) within its intervening sequence.

**Figure 2.**
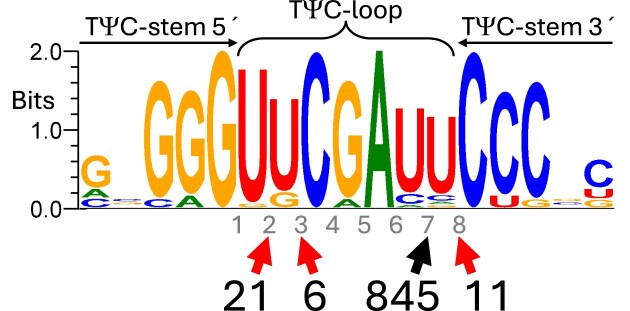
Four positions where group I introns interrupt the tmRNA TψC-loop. A sequence logo for the equally weighted 27 unique post-splicing/pre-modification TψC-stem/loop sequences used by group I introns is shown, with internucleotide loop positions marked with small numbers 1–8. The newly discovered intron positions are 2, 3 and 8. The number of unique pre-tmRNA sequences with an intron at that position is given in large font.

Numerous new genes containing group I introns (883 unique sequences total) were identified, all with the intron in the TψC-loop. The usual rules for group I intron boundary determination are that the preceding (−1) position is a U that pairs with an internal G in the intron P1 stem and the 3′-terminal (ω) position is a G [32]. Boundaries following these rules and respecting reconstitution of the TψC-loop were found in each case, except for 15 sequences where the −1 position is C, paired with A in P1. These boundary rules showed that most had the intron at the previously known [11] T-loop position 7, but that smaller numbers of introns were located at three novel subsites within the TψC-loop (Fig. 2). These four loop subsites are pre-adapted as intron sites because each would most frequently provide a potential −1U. The taxonomic distribution is greatly expanded for those at the main position 7; although still predominantly occurring among the Bacillota, they are now found in 21 different phyla. In contrast, the groups at the minor subsites have limited taxonomic distributions; all those at position 8 are from the family Saprospiraceae, all those at position 2 are from the class Anaerolineae (except for one from the phylum Myxococcota), and all those at position 3 are from the class Bacteroidia. Discovery of new intron sites may be biased by the particular ease of recognizing those in the TψC-loop; we may have systematically missed any in other sites.

A relatively small fraction, 23 of 844, of the founding intron-form sequences contained a homing endonuclease gene (HEG); 4 HEGs were inserted after intron stem P9 and 19 were inserted in the P7.1 region. Our search method may have been biased toward shorter HEG-lacking introns. We prepared an alignment of intron-form sequences and a novel CM (tmRNA_intron) for this gene type. To test whether this CM could find introns with large HEG genes, the 844 founding intron sequences were modified with insertions of random sequence up to 2 kb at either of the two HEG sites; the CM performed well, returning hits spanning the full tmRNA plus intron sequence for 843 of the 844 sequences with 2-kb insertions. The CM also performed well on introns integrated at each of the four tmRNA TψC-loop subsites, perhaps due to the proximity of the subsites. During the attendant sequence alignment, we rejected one founding sequence (VBNU01000423.1/1765-2369) upon observing that it had suffered a deletion or misassembly artifact removing the key intron stem P3. Applying the tmRNA_intron CM to the 33 537 bacterial genomes where the tmRNA gene had not yet been identified, 40 new intron-form gene sequences were discovered, with their group I intron lengths averaging 414 bp, and including the second longest known tmRNA gene sequence at 1116 bp. (The longest at 1152 bp is the permuted HGM16780__sp944390355.1 whose intervening sequence contains a repetitive element.) In contrast, the introns in the 843 founding sequences had a substantially shorter average length of 293 bp. This improved detection of longer intron forms demonstrates the utility of the CM and also shows that the manual phase of our major batch searches may have been biased against longer intron forms. However, the fraction of HEG-bearing intron forms remains low, 36 of 883 (4.1%), compared to 54% for archaeal ribosomal RNA group I introns [33]. None of the discovered introns were longer than 700 bp, despite the ability of the CM to detect introns with simulated HEG insertions of over 2000 bp. The new CM also usually identifies intron genes in their entirety, which previous CMs did not.

**Figure 3.**
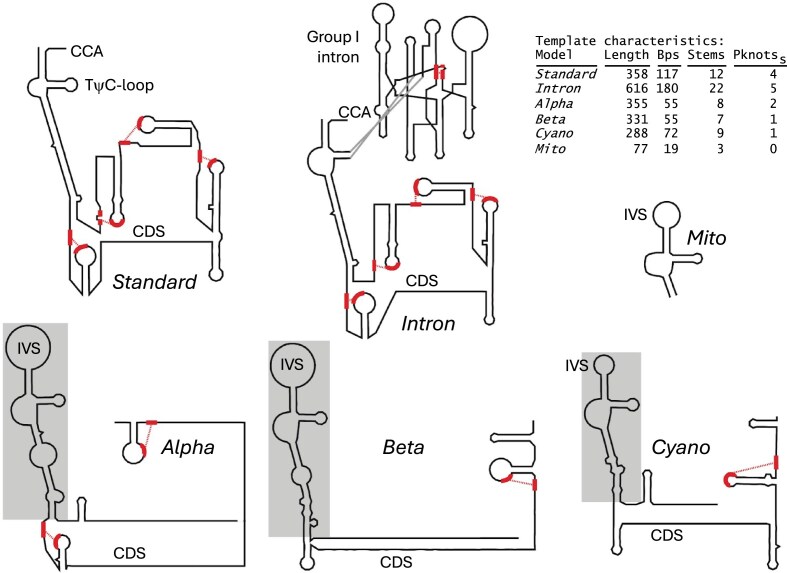
The six tmRNA secondary structure templates, generated by R2DT in “thumbnail” format using the consensus sequence for each template’s CM. Pseudoknot stems not modeled by the CM are highlighted, with each half connected by dashed lines. The boxed regions in the alpha, beta, and cyano templates are the tRNA domains, which include all base pairs modeled by the new, more general tmRNA_permuted Rfam model. Counts of base pairs (Bps) and stems in the table include pseudoknots.

In addition to the novel CM described earlier, we have updated the Rfam CM RF00023 for the standard form that increases bit scores for the standard tmRNAs in our database by 27% compared to the preceding version of the CM. We have also produced a single CM for the permuted form, tmRNA_permuted (newly added to Rfam as CM RF04321), which can be compared to the three original Rfam permuted-form CMs (RF01849, RF01850, and RF01851) that were each designed for one of the three main bacterial clades with permuted forms [8, 31]. The new tmRNA_permuted CM models only the tRNA-like domain, whereas the three Rfam CMs additionally model idiosyncratic sequence and secondary structure for each clade (see Fig. 3). Thus, the best-scoring older Rfam CM on average produces a higher bit score than does tmRNA_permuted. The older Rfam CMs may also find utility in subtyping permuted tmRNA genes, and in finer delimitation of gene termini; moreover, they improve secondary structure rendition by R2DT. Importantly, tmRNA_permuted retrieves all the permuted tmRNAs in our database, while none of the Rfam CMs do. We therefore recommend use of tmRNA_permuted alone for faster and more convenient first-pass permuted tmRNA gene search, followed by application of the three Rfam CMs on each hit if further refinement is required.

We have not modified the Rfam CM RF02544, which is specialized for the usually permuted mitochondrial tmRNA gene [9], but also retrieves the “re-permuted” mitochondrial tmRNA gene of *Jakoba libera* [4]. For comprehensive mitochondrial genome annotation, users may favor the MFannot system [29] that employs RF02544 for tmRNA identification. Through our application of the CM, we report the first identification of tmRNA (Paralagenidium__karlingii.1) in the newly described eukaryotic supergroup Provora [34]. Mitochondrial tmRNA does not appear to have a tag reading frame. It may therefore be unable to perform full *trans*-translation by releasing the ribosome at its tag CDS stop codon. Yet, its conservation (though limited) and co-occurrence with nucleus-encoded mitochondrion-targeted SmpB suggests that it nonetheless helps these cells deal with incomplete mitochondrial mRNAs. We now report apparently CDS-less tmRNAs among plastids, two gene sequences from the Mallomonadaceae that are only ∼65 bp; these show closer homology to other Ochrophyta plastid tmRNAs than to tRNAs. A similar form from phage DS6A (Phage__Mycobacterium.9) has previously been reported, and shown to inhibit the endogenous tmRNA when expressed in *Escherichia coli* [5]. Two bacterial groups may lack tag reading frames (*Nasuia* and 9FT-COMBO-53-11 sp001772805). Aside from these groups that lack or may lack tag CDSs, we now report 25 sequences (e.g. Fermentibacter__daniensis.1) encoding extremely short tag sequences, only 2 aa (Ala–Ala), as compared to the most frequent tag length, 10 aa. This 2-aa tag is especially common among the Fermentibacterota.

To visualize the predicted secondary structure of each database sequence in the canonical tmRNA layout, we developed six templates for use with the R2DT RNA visualization software and used R2DT [15] to produce a secondary structure diagram image for each of the 97 179 sequences. One template corresponds to the new RF00023 standard tmRNA model, one to the new tmRNA_intron model, and three others to the Rfam 15.0 alpha_tmRNA, beta_tmRNA, and cyano_tmRNA models. We reverted to the latter three existing Rfam models instead of using a single template for our new tmRNA_permuted model because of the additional structural information available in each of the Rfam 15.0 models [14], which may be relevant to users examining individual sequences and structures. Example diagrams for each of the five templates are shown in Fig. 3. Structure diagrams for all other database sequences can be viewed and downloaded from the RNAcentral database. The five tmRNA templates have been added as a new library of templates in the R2DT software as of version 2.1, meaning that R2DT tmRNA output diagrams will now adopt the canonical tmRNA layout.

## Conclusions

The greatly enlarged database and new tools for search and visualization will serve many areas of bioinformatics, RNA science, and bacteriology. Group I introns had previously been found in tmRNA in only two bacterial phyla and at only one position in the TψC-loop; they are now found in 21 phyla and at four positions within the TψC-loop. For improved tmRNA gene search in bacterial genomes, we recommend use of BLASTN against our database, Aragorn v. 1.2.40, and the three CMs of Supplementary File S1 (tmRNA, tmRNA_permuted, and tmRNA_intron), combined with tRNAscan-SE v. 2.0 to rule out the most common class of false positives. The new standard- and permuted-form CMs developed here will be included in the next release of Rfam, and so will eventually be used in all Rfam-based genome annotation pipelines, including those employed in databases at the European Bioinformatics Institute, such as Ensembl Genomes [35], and at NCBI, such as PGAP [36].

## Supplementary Material

lqaf019_Supplemental_Files

## Data Availability

The database of tmRNA sequences and metadata can be retrieved as a flat file at http://doi.org/10.6084/m9.figshare.28430909, and data are available via the API, FTP archive, and other interfaces from RNAcentral (https://rnacentral.org/expert-database/tmrna-website) [37]. The new CMs are available as Supplementary File S1. The new R2DT templates are available as part of the R2DT v. 2.1 release.
