# Supplementary material for: Expansion of the tmRNA sequence database and new tools for search and visualization
Source: NAR Genom Bioinform. 2025 Mar 18;7(1):lqaf019. doi: 10.1093/nargab/lqaf019 (PMC11915505; doi:10.1093/nargab/lqaf019)
Supplement: lqaf019_Supplemental_Files [file lqaf019_supplemental_files.zip › SuppMatls.docx]

Supplementary Materials List

Supplementary Table 1. Genomes treated (see Materials and Methods). Genomes without tmRNA matches are identified using the 9-digit portion of the GenBank assembly IDs. For the 63 Archaea and 418144 Bacteria genomes with tmRNA matches, see Database file tmrnaDatabase.tsv (http://doi.org/10.6084/m9.figshare.28430909).

Supplementary Table 2. Phylum-level breakdown of archaeal and bacterial occurrences of the various tmRNA gene forms. Counts of instances are reported rather than unique sequence counts, because some unique sequences cross phylum or even domain boundaries. Parenthetical note after “Permuted” header: the best-scoring Rfam CM. Parenthetical note after “Intron” header: position of the intron in the TψC-loop (Figure 2).

Supplementary File 1. Three new or updated covariation models and the alignments used to prepare them: tmRNA (update to Rfam RF00023), tmRNA_permuted (new Rfam RF04321), tmRNA_intron.

Database File tsv (http://doi.org/10.6084/m9.figshare.28430909). Unique tmRNA sequences. Columns:

1. Unique ID
2. Gene form
3. Gene segments and their positions on the segments
4. Encoded proteolytic tag sequence
5. Genetic code used for tag translation (https://en.wikipedia.org/wiki/List_of_genetic_codes)
6. Comma-separated scores from Aragorn v1.2.40 and the CMs alpha_tmRNA, beta_tmRNA, cyano_tmRNA, permuted_tmRNA, mt-tmRNA, tmRNA (old version), tmRNA (new version), tmRNA_intron
7. Taxonomy (for bacteria/archaea, from GTDB release 214, otherwise from NCBI)
8. Gene sequence with lower case marking tag CDS, intervening sequence (IVS), and bases corresponding to CCA tail
9. Count of known instances of this sequence
10. Known instances ([assembly ID/]accession/coordinates)
11. Search phase (see Methods)
12. Note
